# Supplementary material for: Adolescent wellbeing is associated with positive outcomes in early adulthood in a sibling comparison study
Source: Nat Commun. 2026 May 11;17:4109. doi: 10.1038/s41467-026-72459-9 (PMC13161430; doi:10.1038/s41467-026-72459-9)
Supplement: Supplementary file 1 — Supplementary Materials [file 41467_2026_72459_MOESM1_ESM.pdf]

Supplementary Information

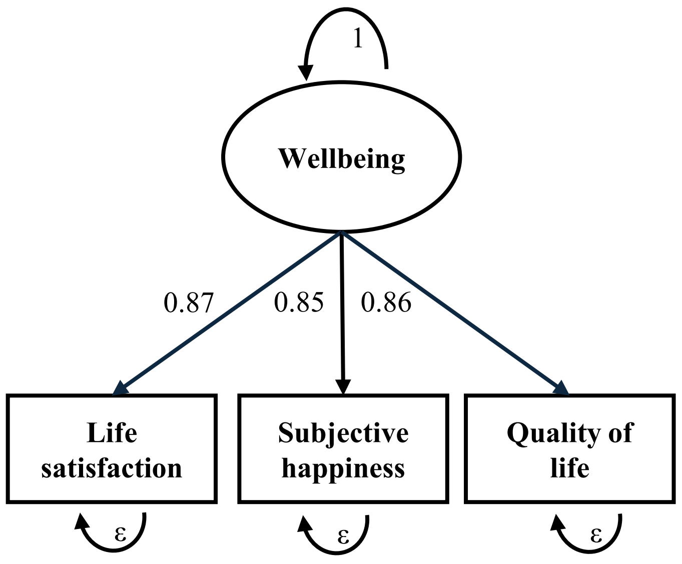

Supplementary Figure S1. A schematic path diagram of the measurement model of adolescent and early adulthood wellbeing, estimated using confirmatory factor analysis (CFA). Wellbeing is modelled as a latent factor indicated by life satisfaction, subjective happiness, and quality of life. ε represents the residual variance of the observed variable. Loadings shown are from survey 14.

Supplementary Table S1. Number of observations for each exposure and outcome variable

	14 years (n=10880)		16 years (n=7492)		14-16 years (n=14518)	
**Age**	20-25	25-35	20-25	25-35	20-25	25-35
**Wellbeing outcomes**						
Wellbeing score	2679	1180	2638	1491	3810	1872
Flourishing score	1187	1162	724	1295	1364	1649
**Social outcomes**						
Personality traits	1916	149	2291	504	2923	570
**Demographic outcomes**						
Children (yes/no)	2843	1287	2754	1605	4026	2038
Stable relationship (yes/no)	2895	1294	2788	1600	4087	2037
Employment (yes/no)	2643	1074	2644	1423	3754	1757
Education, diploma (yes/no)	3694	1500	3658	2006	5157	2483
**Health outcomes**						
Health status (illnesses at the moment yes/no)	127	42	566	158	610	176
Self-rated health	3713	1382	3594	1896	5161	2349
BMI (kg/m^e)	4201	1631	4052	2121	5859	2664
**Lifestyle outcomes**						
Alcohol use	994	82	1339	294	1689	334
Coffee use (cups/day)	579	58	655	210	868	237
Drug use	1915	147	2269	506	2905	572
Smoking cigarettes (never/in the past/current)	4195	1670	3972	2164	5774	2723
Smoking e-cigarettes (yes/no)	1722	137	1890	491	2422	549
Smoking hash, marijuana (yes/no)	1871	1203	1738	1360	2704	1762
Physical activity (sedentary/moderate/vigorous)	3200	1339	3095	1669	4558	2127
Sleep duration (h)	113	8	518	24	554	29
Sleep quality	2424	1014	2502	1346	3483	1661
Eating behaviour	1461	103	1783	348	2232	390
Dieting	462	25	798	33	989	50
Weight behaviour (gaining weight)	462	26	798	32	990	50

Supplementary Table S2 Correlations between the two-measure wellbeing factor scores, i.e. survey 10, vs. the three-measure wellbeing factor scores, i.e. survey 8 and 14

	**Survey 8**	**Survey 10**	**Survey 14**
**Survey 8**	1	0.62	0.55
**Survey 10**	0.62	1	0.62
**Survey 14**	0.55	0.62	1

Supplementary Table S3. Between-family GEE regression models between adolescent wellbeing and outcome meaures at age 20-25. Results are based on two-sided tests; p values were adjusted for multiple testing using the Benjamini–Hochberg false discovery rate (FDR) procedure. Exact FDR-adjusted p-values and explained variance percentages are shown.

Between-family | Age 20-25	Model 1										Model 2									
Outcome	n	StBeta	p-value	pFDR	SE	lowerCI	upperCI	R2	R2null	Rincr	n	StBeta	p-value	pFDR	SE	lowerCI	upperCI	R2	R2null	Rincr
Wellbeing	3810	0,38	0,00000	0,0000000	0,02	0,34	0,42	0,14	0,00	13,7%										
Flourishing	1364	0,34	0,00000	0,0000000	0,03	0,28	0,41	0,12	0,00	11,4%										
Personality - Neuroticism	2923	-0,34	0,00000	0,0000000	0,02	-0,38	-0,30	0,16	0,05	10,9%	1152	-0,11	0,00001	0,000076	0,03	-0,16	-0,06	0,18	0,18	0,3%
Personality - Extraversion	2923	0,25	0,00000	0,0000000	0,02	0,21	0,29	0,06	0,00	5,9%	1152	0,05	0,11305	0,195261	0,03	-0,01	0,10	0,17	0,17	0,1%
Personality - Openness	2923	-0,05	0,01259	0,0214099	0,02	-0,09	-0,01	0,01	0,00	0,2%	1152	-0,01	0,77993	0,875754	0,03	-0,06	0,04	0,16	0,16	0,0%
Personality - Agreeableness	2923	0,09	0,00000	0,0000113	0,02	0,05	0,13	0,08	0,07	0,8%	1152	-0,04	0,09213	0,175052	0,02	-0,09	0,01	0,14	0,14	0,1%
Personality - Conscientiousness	2923	0,22	0,00000	0,0000000	0,02	0,18	0,26	0,06	0,02	4,5%	1152	0,08	0,00176	0,008377	0,03	0,03	0,13	0,16	0,15	0,2%
Self-rated health	5161	0,18	0,00000	0,0000000	0,01	0,15	0,21	0,05	0,02	3,2%	5135	0,09	0,00000	0,000000	0,02	0,06	0,12	0,10	0,10	0,7%
BMI	5859	-0,07	0,00003	0,0000972	0,02	-0,10	-0,04	0,01	0,01	0,5%	5701	-0,04	0,00339	0,012097	0,01	-0,06	-0,01	0,47	0,46	0,1%
Coffee use	868	-0,07	0,02165	0,0350503	0,03	-0,13	-0,01	0,10	0,09	0,5%										
Sleep duration	554	0,02	0,62489	0,6853624	0,04	-0,06	0,09	0,06	0,06	0,0%	551	-0,01	0,78357	0,8757542	0,04	-0,08	0,06	0,11	0,11	0,0%
**Children**																				
No	3948																			
Yes	78	-0,51	0,50536	0,5727867	0,76	-2,00	0,98	0,01	0,01	0,0%										
**Stable relationship**																				
No	2726																			
Yes	1361	0,02	0,78776	0,8370003	0,08	-0,13	0,17	0,09	0,09	0,0%										
**Working**																				
No	1650										1646									
Yes	2258	0,01	0,89112	0,8911172	0,07	-0,12	0,14	0,05	0,05	0,0%	2241	-0,01	0,89565	0,895650	0,07	-0,15	0,13	0,12	0,12	0,0%
**Diploma**																				
No	1420										1416									
Yes	3737	-0,01	0,86855	0,8911172	0,08	-0,16	0,14	0,08	0,08	0,0%	3714	-0,01	0,87343	0,895650	0,08	-0,16	0,14	0,09	0,09	0,0%
**Health status (illnesses)**																				
No	400										382									
Yes	210	-0,15	0,41950	0,5093927	0,18	-0,51	0,21	0,02	0,01	0,1%	206	-0,14	0,48639	0,680268	0,20	-0,52	0,25	0,09	0,09	0,1%
**Alcohol use**	1689	-0,06	0,09055	0,1282792	0,03	-0,12	0,01	0,12	0,12	0,1%	1155	-0,04	0,34531	0,546741	0,04	-0,11	0,04	0,21	0,21	0,1%
**Drug use**																				
No	2541																			
Yes	364	-0,30	0,08008	0,1183806	0,17	-0,64	0,04	0,01	0,01	0,1%										
**Smoking**																				
Never	3268	0,10	0,00489	0,0092367	0,04	0,03	0,17				3251	0,07	0,05795	0,122339	0,04	0,00	0,14			
In the past	864	-0,03	0,50540	0,572787	0,05	-0,12	0,06				852	-0,03	0,50125	0,680268	0,05	-0,12	0,06			
Current	1642							0,02	0,01	0,3%	1624							0,08	0,08	0,1%
**Smoking e-cigarettes**																				
No	2219																			
Yes	203	-0,48	0,05760	0,089020	0,25	-0,98	0,02	0,00	0,00	0,2%										
**Smoking hash, marijuana**																				
No	1761										1095									
Yes	943	-0,33	0,00011	0,000343	0,09	-0,50	-0,16	0,04	0,03	0,5%	613	-0,29	0,00780	0,021158	0,11	-0,50	-0,08	0,07	0,07	0,4%
**Physical activity**																				
Sedentary	1777	-0,27	0,00001	0,0000378	0,06	-0,39	-0,15				1774	-0,18	0,00382	0,012097	0,06	-0,30	-0,06			
Moderate	1875	-0,08	0,17517	0,2205844	0,06	-0,20	0,04				1872	-0,02	0,69691	0,875754	0,06	-0,15	0,10			
Vigorous	906							0,05	0,04	0,9%	904							0,16	0,16	0,4%
**Sleep quality**																				
No trouble sleeping	2555	0,87	0,00000	0,0000000	0,09	0,68	1,05				2545	0,59	0,00000	0,000000	0,11	0,38	0,79			
Some trouble sleeping	678	0,34	0,00032	0,0009067	0,10	0,16	0,53				677	0,21	0,05097	0,121054	0,11	0,00	0,41			
Often trouble sleeping	250							0,06	0,02	4,3%	249							0,14	0,12	1,7%
**Eating behaviour**																				
Stops eating before full	398	0,41	0,00693	0,012401	0,15	0,11	0,70													
Stops eating when full	1612	0,43	0,00042	0,0010985	0,12	0,19	0,66													
Continues eating when full	222							0,02	0,01	0,7%										
**Dieting**											computational singularity									
Never went on a diet	684	0,49	0,00099	0,0021038	0,15	0,20	0,78				computational singularity									
Went on a diet a couple of times	208	0,50	0,00353	0,007060	0,17	0,16	0,84				computational singularity									
Went on a diet several times	49	0,29	0,11551	0,1510515	0,19	-0,07	0,66				computational singularity									
Often or always on a diet	48							0,14	0,13	1,2%	computational singularity									
**Weight behaviour**											computational singularity									
Not afraid to gain weight	377	0,37	0,00086	0,001949	0,11	0,15	0,59				computational singularity									
Somewhat afraid to gain weight	378	0,37	0,00051	0,0012386	0,11	0,16	0,57				computational singularity									
Quite afraid to gain weight	141	0,19	0,11068	0,1505248	0,12	-0,04	0,43				computational singularity									
Very afraid to gain weight	94							0,20	0,19	1,3%	computational singularity									

Supplementary Table S4. Between-family GEE regression models between adolescent wellbeing and outcome meaures at age 25-35. Results are based on two-sided tests; p values were adjusted for multiple testing using the Benjamini–Hochberg false discovery rate (FDR) procedure. Exact FDR-adjusted p-values and explained variance percentages are shown.

Between-family | Age 25-35	Model 1										Model 2									
Outcome	n	StBeta	p-value	pFDR	SE	lowerCI	upperCI	R2	R2null	Rincr	n	StBeta	p-value	pFDR	SE	lowerCI	upperCI	R2	R2null	Rincr
Wellbeing	1872	0,32	0,00000	0,0000000	0,03	0,27	0,37	0,10	0,00	10,1%										
Flourishing	1649	0,27	0,00000	0,0000000	0,03	0,21	0,33	0,07	0,00	6,9%										
Personality - Neuroticism	570	-0,34	0,00000	0,0000000	0,04	-0,42	-0,27	0,17	0,05	12,6%	88	-0,37	0,00064	0,0040658	0,11	-0,58	-0,16	0,08	0,07	1,0%
Personality - Extraversion	570	0,24	0,00000	0,0000006	0,04	0,15	0,32	0,06	0,00	5,9%	88	0,05	0,52447	0,6228117	0,08	-0,11	0,22	0,07	0,07	0,0%
Personality - Openness	570	-0,04	0,28473	0,3466324	0,04	-0,13	0,04	0,00	0,00	0,2%	88	-0,11	0,38225	0,4841867	0,13	-0,36	0,14	0,05	0,05	0,1%
Personality - Agreeableness	570	0,10	0,01461	0,0371995	0,04	0,02	0,17	0,08	0,07	1,0%	88	0,16	0,10883	0,2297535	0,10	-0,04	0,35	0,08	0,08	0,3%
Personality - Conscientiousness	570	0,18	0,00000	0,0000126	0,04	0,11	0,26	0,05	0,02	3,6%	88	0,20	0,01475	0,0560505	0,08	0,04	0,37	0,06	0,06	0,4%
Self-rated health	2349	0,18	0,00000	0,0000000	0,02	0,13	0,22	0,04	0,01	3,1%	2342	0,09	0,00020	0,0018710	0,02	0,04	0,13	0,10	0,10	0,7%
BMI	2664	-0,08	0,00033	0,0010387	0,02	-0,12	-0,04	0,01	0,01	0,6%	2575	-0,03	0,09257	0,2297535	0,02	-0,06	0,00	0,43	0,43	0,1%
Coffee use	237	-0,09	0,10061	0,1657030	0,05	-0,19	0,02	0,11	0,10	0,8%										
Sleep duration	29	-0,23	0,26240	0,3339617	0,21	-0,63	0,17	0,31	0,28	3,6%	29	-0,20	0,34062	0,4622704	0,21	-0,60	0,21	0,34	0,32	2,6%
**Children**																				
No	1691																			
Yes	347	-0,27	0,14280	0,2221383	0,18	-0,62	0,09	0,15	0,15	0,1%										
**Stable relationship**																				
No	653																			
Yes	1384	0,14	0,17349	0,2556673	0,10	-0,06	0,35	0,03	0,03	0,1%										
**Working**																				
No	903										903									
Yes	835	-0,04	0,73821	0,7655545	0,12	-0,27	0,19	0,30	0,30	0,0%	829	-0,01	0,95719	0,9571850	0,19	-0,39	0,37	0,72	0,72	0,0%
**Diploma**																				
No	297										295									
Yes	2186	0,26	0,19569	0,2739623	0,20	-0,13	0,65	0,01	0,01	0,1%	2181	0,25	0,21914	0,3678254	0,20	-0,15	0,65	0,02	0,02	0,1%
**Health status (illnesses)**																				
No	81										78									
Yes	95	-0,38	0,23428	0,3123681	0,32	-1,01	0,25	0,05	0,04	0,8%	91	-0,48	0,15351	0,2916777	0,34	-1,15	0,18	0,10	0,08	1,0%
**Alcohol use**	334	0,02	0,80131	0,8013100	0,08	-0,14	0,18	0,10	0,10	0,0%	282	0,02	0,78969	0,8825947	0,09	-0,15	0,20	0,21	0,21	0,0%
**Drug use**																				
No	491																			
Yes	81	0,20	0,58156	0,6513522	0,37	-0,52	0,92	0,01	0,00	0,1%										
**Smoking**																				
Never	1797	0,15	0,02689	0,0579169	0,07	0,02	0,28				1780	0,08	0,25167	0,3678254	0,07	-0,06	0,22			
In the past	458	-0,04	0,62359	0,6715585	0,08	-0,21	0,12				456	-0,01	0,90511	0,9553939	0,09	-0,18	0,16			
Current	468							0,08	0,08	0,4%	458							0,23	0,23	0,1%
**Smoking e-cigarettes**																				
No	506																			
Yes	43	-0,82	0,04983	0,0930129	0,42	-1,63	0,00	0,01	0,01	0,5%										
**Smoking hash, marijuana**																				
No	1144										627									
Yes	618	-0,36	0,00118	0,0032949	0,11	-0,57	-0,14	0,04	0,03	0,6%	351	-0,32	0,03447	0,1091675	0,15	-0,62	-0,02	0,13	0,13	0,4%
**Physical activity**																				
Sedentary	1002	-0,42	0,00009	0,0003150	0,11	-0,63	-0,21				1001	-0,34	0,00153	0,0072675	0,11	-0,56	-0,13			
Moderate	769	-0,24	0,03217	0,0643400	0,11	-0,46	-0,02				769	-0,18	0,10806	0,2297535	0,11	-0,40	0,04	0,11	0,10	0,8%
Vigorous	356							0,04	0,03	1,3%	355									
**Sleep quality**																				
No trouble sleeping	1146	0,85	0,00000	0,0000000	0,12	0,63	1,08				1143	0,66	0,00000	0,0000000	0,12	0,41	0,90			
Some trouble sleeping	365	0,21	0,05983	0,1047025	0,11	-0,01	0,44				365	0,14	0,24267	0,3678254	0,12	-0,10	0,38			
Often trouble sleeping	150							0,06	0,01	5,8%	150							0,13	0,11	2,9%
**Eating behaviour**																				
Stops eating before full	88	0,31	0,34029	0,3970050	0,33	-0,33	0,95													
Stops eating when full	267	0,67	0,01895	0,0442167	0,29	0,11	1,23													
Continues eating when full	35							0,03	0,01	2,0%										
**Dieting**	computational singularity										computational singularity									
Never went on a diet	computational singularity										computational singularity									
Went on a diet a couple of times	computational singularity										computational singularity									
Went on a diet several times	computational singularity										computational singularity									
Often or always on a diet	computational singularity										computational singularity									
**Weight behaviour**	computational singularity										computational singularity									
Not afraid to gain weight	computational singularity										computational singularity									
Somewhat afraid to gain weight	computational singularity										computational singularity									
Quite afraid to gain weight	computational singularity										computational singularity									
Very afraid to gain weight	computational singularity										computational singularity									

Supplementary Table S5. Within-family GEE regression models between adolescent wellbeing and outcome meaures at age 20-25. Results are based on two-sided tests; p values were adjusted for multiple testing using the Benjamini–Hochberg false discovery rate (FDR) procedure. Exact FDR-adjusted p-values and explained variance percentages are shown.

Within-family | Age 20-25	Model 1										Model 2									
Outcome	n	StBeta	p-value	pFDR	SE	lowerCI	upperCI	R2	R2null	Rincr	n	StBeta	p-value	pFDR	SE	lowerCI	upperCI	R2	R2null	Rincr
Wellbeing	3589	0,12	0,00023	0,00463	0,03	0,06	0,19	0,14	0,14	0,0%										
Flourishing	1236	0,17	0,00209	0,01305	0,06	0,06	0,28	0,10	0,10	0,0%										
Personality - Neuroticism	2807	-0,12	0,00037	0,00463	0,03	-0,19	-0,05	0,15	0,15	0,0%	1098	-0,04	0,29727	0,5994494	0,04	-0,12	0,04	0,17	0,17	0,0%
Personality - Extraversion	2807	0,07	0,04517	0,16134	0,03	0,00	0,13	0,06	0,06	0,0%	1098	0,03	0,61459	0,6325600	0,06	-0,08	0,14	0,16	0,16	0,0%
Personality - Openness	2807	-0,03	0,40731	0,72734	0,03	-0,10	0,04	0,01	0,01	0,0%	1098	-0,04	0,39839	0,6325600	0,05	-0,14	0,06	0,16	0,16	0,0%
Personality - Agreeableness	2807	0,00	0,98269	0,98269	0,03	-0,06	0,06	0,08	0,08	0,0%	1098	-0,05	0,21415	0,5994494	0,04	-0,12	0,03	0,13	0,13	0,0%
Personality - Conscientiousness	2807	0,07	0,03534	0,14725	0,04	0,01	0,14	0,07	0,07	0,0%	1098	0,05	0,29972	0,5994494	0,05	-0,05	0,16	0,15	0,15	0,0%
Self-rated health	4823	0,07	0,00601	0,03006	0,03	0,02	0,12	0,04	0,04	0,0%	4800	0,03	0,18164	0,5994494	0,03	-0,02	0,09	0,09	0,09	0,0%
BMI	5410	-0,01	0,51270	0,82463	0,02	-0,06	0,03	0,01	0,01	0,0%	5264	-0,02	0,26831	0,5994494	0,02	-0,05	0,02	0,44	0,44	0,0%
Coffee use	829	0,08	0,21095	0,52736	0,06	-0,05	0,21	0,10	0,10	0,0%										
Sleep duration																				
**Children**																				
No																				
Yes																				
**Stable relationship**																				
No																				
Yes																				
**Working**																				
No																				
Yes																				
**Diploma**																				
No																				
Yes																				
**Health status (illnesses)**																				
No																				
Yes																				
**Alcohol use**																				
**Drug use**																				
No																				
Yes																				
**Smoking**																				
Never	3020	0,06	0,26003	0,590977	0,06	-0,05	0,18				3020	0,06	0,29204	0,599449	0,06	-0,06	0,18			
In the past	806	0,05	0,64197	0,833200	0,10	-0,16	0,25				806	0,06	0,56480	0,632560	0,10	-0,14	0,26			
Current	1519							0,02	0,02	0,0%	1519							0,09	0,09	0,0%
**Smoking e-cigarettes**																				
No																				
Yes																				
**Smoking hash, marijuana**																				
No	865										865									
Yes	1661	-0,06	0,72533	0,863485	0,16	-0,37	0,26	0,04	0,04	0,0%	1661	-0,12	0,58272	0,632560	0,22	-0,54	0,31	0,05	0,05	0,0%
**Physical activity**																				
Sedentary	1645	-0,09	0,37443	0,727337	0,10	-0,28	0,11				1645	-0,06	0,55312	0,632560	0,11	-0,27	0,15			
Moderate	1783	-0,07	0,52776	0,824625	0,11	-0,28	0,14				1783	-0,05	0,63256	0,632560	0,11	-0,26	0,16			
Vigorous	856							0,05	0,05	0,0%	856							0,16	0,16	0,0%
**Sleep quality**																				
No trouble sleeping	2437	0,46	0,00163	0,013054	0,15	0,17	0,75				2437	0,27	0,11145	0,599449	0,17	-0,06	0,61			
Some trouble sleeping	635	0,23	0,17305	0,523278	0,17	-0,10	0,55				635	0,12	0,51252	0,632560	0,18	-0,23	0,47			
Often trouble sleeping	228							0,06	0,06	0,0%	228							0,14	0,14	0,0%
**Eating behaviour**																				
Stops eating before full	389	0,07	0,83455	0,869323	0,31	-0,55	0,68													
Stops eating when full	1556	0,13	0,62383	0,833200	0,27	-0,40	0,67													
Continues eating when full	210							0,02	0,02	0,0%										
**Dieting**											computational singularity									
Never went on a diet	660	0,12	0,80319	0,869323	0,46	-0,79	1,02				computational singularity									
Went on a diet a couple of times	206	0,25	0,65350	0,833200	0,56	-0,85	1,35				computational singularity									
Went on a diet several times	47	0,51	0,39486	0,727337	0,61	-0,67	1,70				computational singularity									
Often or always on a diet	47							0,14	0,14	0,0%	computational singularity									
**Weight behaviour**											computational singularity									
Not afraid to gain weight	366	-0,13	0,66656	0,833200	0,29	-0,70	0,45				computational singularity									
Somewhat afraid to gain weight	369	0,09	0,77094	0,869323	0,31	-0,51	0,69				computational singularity									
Quite afraid to gain weight	136	-0,45	0,18838	0,523278	0,34	-1,12	0,22				computational singularity									
Very afraid to gain weight	90							0,21	0,21	0,0%	computational singularity									

Supplementary Table S6. Within-family GEE regression models between adolescent wellbeing and outcome meaures at age 25-35. Results are based on two-sided tests; p values were adjusted for multiple testing using the Benjamini–Hochberg false discovery rate (FDR) procedure. Exact FDR-adjusted p-values and explained variance percentages are shown.

Within-family | Age 25-35	Model 1										Model 2									
Outcome	n	StBeta	p-value	pFDR	SE	lowerCI	upperCI	R2	R2null	Rincr	n	StBeta	p-value	pFDR	SE	lowerCI	upperCI	R2	R2null	Rincr
Wellbeing	1776	0,12	0,01207	0,090514	0,05	0,03	0,22	0,10	0,10	0,0%										
Flourishing	1554	0,09	0,08252	0,242276	0,05	-0,01	0,19	0,07	0,07	0,0%										
Personality - Neuroticism	559	-0,18	0,05449	0,204346	0,09	-0,36	0,00	0,17	0,17	0,0%	computational singularity									
Personality - Extraversion	559	0,01	0,83301	0,833007	0,06	-0,11	0,14	0,07	0,07	0,0%	computational singularity									
Personality - Openness																				
Personality - Agreeableness	559	0,02	0,82597	0,833007	0,08	-0,14	0,17	0,08	0,08	0,0%	computational singularity									
Personality - Conscientiousness	559	0,17	0,09691	0,242276	0,10	-0,03	0,36	0,06	0,06	0,0%	computational singularity									
Self-rated health	2230	0,05	0,17638	0,377963	0,04	-0,02	0,12	0,04	0,04	0,0%	2225	0,02	0,58239	0,582393	0,04	-0,05	0,09	0,10	0,10	0,0%
BMI	2541	-0,03	0,26095	0,425760	0,03	-0,09	0,02	0,01	0,01	0,0%	2459	-0,02	0,49979	0,582393	0,02	-0,06	0,03	0,41	0,41	0,0%
Coffee use																				
Sleep duration																				
**Children**																				
No																				
Yes																				
**Stable relationship**																				
No																				
Yes																				
**Working**																				
No																				
Yes																				
**Diploma**																				
No																				
Yes																				
**Health status (illnesses)**																				
No																				
Yes																				
**Alcohol use**																				
**Drug use**																				
No																				
Yes																				
**Smoking**																				
Never																				
In the past																				
Current																				
**Smoking e-cigarettes**																				
No																				
Yes																				
**Smoking hash, marijuana**																				
No	1088										1088									
Yes	579	-0,43	0,02534	0,126676	0,19	-0,80	-0,05	0,04	0,04	0,0%	579	-0,38	0,13716	0,44534	0,25	-0,87	0,12	0,07	0,07	0,0%
**Physical activity**																				
Sedentary	943	0,12	0,46079	0,628350	0,16	-0,20	0,44				943	0,14	0,40584	0,56843	0,17	-0,20	0,49			
Moderate	742	0,21	0,22967	0,425760	0,18	-0,13	0,56				742	0,24	0,19086	0,44534	0,18	-0,12	0,59			
Vigorous	338							0,07	0,07	0,0%	338							0,13	0,13	0,0%
**Sleep quality**																				
No trouble sleeping	1101	0,71	0,00103	0,015450	0,22	0,28	1,13				1101	0,60	0,00763	0,05341	0,23	0,16	1,05			
Some trouble sleeping	352	0,26	0,28384	0,425760	0,24	-0,22	0,74				352	0,21	0,40602	0,56843	0,25	-0,28	0,70			
Often trouble sleeping	140							0,07	0,07	0,0%	140							0,13	0,13	0,0%
**Eating behaviour**																				
Stops eating before full	85	-0,44	0,64773	0,747381	0,97	-2,35	1,46													
Stops eating when full	264	-0,33	0,64493	0,747381	0,72	-1,74	1,08													
Continues eating when full	32							0,05	0,05	0,0%										
**Dieting**																				
Never went on a diet																				
Went on a diet a couple of times																				
Went on a diet several times																				
Often or always on a diet																				
**Weight behaviour**																				
Not afraid to gain weight																				
Somewhat afraid to gain weight																				
Quite afraid to gain weight																				
Very afraid to gain weight																				

Supplementary Materials: mediation analysis

Preregistered mediation analyses were conducted to examine whether associations between adolescent wellbeing (exposure) and outcomes at age 25–35 operated through potential mediators measured at age 20–25. Mediation models were estimated using GEEmediate [1], which accounts for family clustering. Consistent with the preregistration, analyses were conducted for the associations that were shown to be significant in the within-family analyses to test how adolescent wellbeing and outcome measures are related through potential direct and indirect effects of a mediator variable [2,3]. Candidate mediators included wellbeing, flourishing, neuroticism, self-rated health, and sleep quality measured at age 20–25. Sleep quality at age 25-35 was the only significant outcome measure in the within-family analysis at age 25-25. Because sleep quality was measured as a categorical variable, mediation analyses involving sleep quality were implemented as pairwise binary contrasts (none vs often; some vs often).

None of the candidate mediators showed evidence of an indirect effect linking adolescent wellbeing to sleep outcomes at age 25–35. Full model estimates are provided in Supplementary Table S7.

References

1. Nevo, D., Liao, X. & Spiegelman, D. Estimation and Inference for the Mediation Proportion. *Int. J. Biostat.* **13**, (2017).
2. Agler, R. & De Boeck, P. On the Interpretation and Use of Mediation: Multiple Perspectives on Mediation Analysis. *Front. Psychol.* **8**, (2017).
3. Rijnhart, J. J. M. *et al.* Mediation analysis methods used in observational research: a scoping review and recommendations. *BMC Med. Res. Methodol.* **21**, 226 (2021).

Table S7. Full model estimates of the mediation analyses

Exposure at age 14-16	Mediator at age 20-25	Outcome at age 25-35	n	Indirect Effect	Lower 95% CI	Upper 95% CI	p value	Direct Effect	Mediation proportion	Lower 95% CI	Upper 95% CI	p value
Adolescent wellbeing	Wellbeing	Sleep quality - None vs often	942	0.26	-0.05	0.58	0.10243	0.35	43.2%	-0.4%	86.7%	0.02600
Adolescent wellbeing	Wellbeing	Sleep quality - Some vs often	384	0.17	-0.18	0.51	0.34474	-0.03	-			
Adolescent wellbeing	Flourishing	Sleep quality - None vs often	256	0.15	-0.54	0.84	0.66611	0.53	22.3%	-69.4%	114.1%	0.31668
Adolescent wellbeing	Flourishing	Sleep quality - Some vs often	124	0.16	-0.42	0.74	0.59190	-0.06	-			
Adolescent wellbeing	Neuroticism	Sleep quality - None vs often	838	0.29	-0.05	0.63	0.09182	0.32	47.4%	1.8%	93.0%	0.02082
Adolescent wellbeing	Neuroticism	Sleep quality - Some vs often	318	0.15	-0.20	0.51	0.39294	-0.02	-			
Adolescent wellbeing	Self-rated health	Sleep quality - None vs often	1120	0.09	-0.19	0.36	0.53365	0.56	13.5%	-26.2%	53.2%	0.25301
Adolescent wellbeing	Self-rated health	Sleep quality - Some vs often	444	0.06	-0.25	0.37	0.69682	0.11	35.2%	-115.1%	185.5%	0.32294
Adolescent wellbeing	Sleep quality - None vs often	Sleep quality - None vs often	846	0.23	-0.21	0.66	0.30337	0.40	36.4%	-26.2%	98.9%	0.12725
Adolescent wellbeing	Sleep quality - Some vs often	Sleep quality - Some vs often	235	-0.05	-0.54	0.44	0.85153	0.16	-			

Indirect effect: natural indirect effect of the exposure on the outcome through the mediator. Direct effect: natural direct effect of the exposure on the outcome not operating through the mediator. Mediation proportion: proportion of the total effect explained by the indirect pathway, calculated as the indirect effect divided by the total effect. Confidence intervals correspond to 95% confidence intervals estimated using the GEEmediate procedure [1]. Note: Mediation proportions are not reported when the indirect and direct effects have opposite signs or when the total effect approaches zero, as the resulting proportions fall outside the interpretable 0–100% range.
