# Supplementary material for: Adolescent wellbeing is associated with positive outcomes in early adulthood in a sibling comparison study
Source: Nat Commun. 2026 May 11;17:4109. doi: 10.1038/s41467-026-72459-9 (PMC13161430; doi:10.1038/s41467-026-72459-9)
Supplement: Supplementary file 2 — Reporting Summary [file 41467_2026_72459_MOESM2_ESM.pdf]

nature portfolio

Corresponding author(s): Anne J.M.R. Geijsen

Last updated by author(s): Feb 9, 2026

Reporting Summary

Nature Portfolio wishes to improve the reproducibility of the work that we publish. This form provides structure for consistency and transparency in reporting. For further information on Nature Portfolio policies, see our Editorial Policies and the Editorial Policy Checklist.

Statistics

For all statistical analyses, confirm that the following items are present in the figure legend, table legend, main text, or Methods section.

n/a	Confirmed	
☒	☐	The exact sample size (n) for each experimental group/condition, given as a discrete number and unit of measurement
☐	☒	A statement on whether measurements were taken from distinct samples or whether the same sample was measured repeatedly
☐	☒	The statistical test(s) used AND whether they are one- or two-sided<br>*Only common tests should be described solely by name; describe more complex techniques in the Methods section.*
☐	☒	A description of all covariates tested
☐	☒	A description of any assumptions or corrections, such as tests of normality and adjustment for multiple comparisons
☐	☒	A full description of the statistical parameters including central tendency (e.g. means) or other basic estimates (e.g. regression coefficient) AND variation (e.g. standard deviation) or associated estimates of uncertainty (e.g. confidence intervals)
☐	☒	For null hypothesis testing, the test statistic (e.g. F, t, r) with confidence intervals, effect sizes, degrees of freedom and P value noted<br>*Give P values as exact values whenever suitable.*
☒	☐	For Bayesian analysis, information on the choice of priors and Markov chain Monte Carlo settings
☒	☐	For hierarchical and complex designs, identification of the appropriate level for tests and full reporting of outcomes
☐	☒	Estimates of effect sizes (e.g. Cohen's d, Pearson's r), indicating how they were calculated

Our web collection on statistics for biologists contains articles on many of the points above.

Software and code

Policy information about availability of computer code

Data collection	Questionnaires were distributed in waves to adolescent twins and their siblings, following parental consent. Every two to three years, NTR participants 18 years and older receive questionnaires to measure various sociodemographic, health, and lifestyle-related factors including BMI, physical activity, smoking habits, and wellbeing. In more recent surveys, questionnaires were surveyed using online survey tools (Survalyzer, NetQ). More information about NTR data collection see 10.1375/twin.5.5.401, 10.107/thg.2019.93, and 10.1017/thg.2012.118
Data analysis	All analyses were performed in R version 4.4.0 (R Core Team, 2024) using the packages foreign (R, Core Team, 2023), lavaan (Rosseel, 2012), dplyr (Hadley Wickham et al., 2023), gee (Vincent J. Carey, 2024), multgee (Touloumis, A. et al., 2015), GEEmediate (Nevo et al., 2017), and forestploter (Alimu Dayimu, 2024). See https://osf.io/7fyx2/overview for R code, including the used packages.

For manuscripts utilizing custom algorithms or software that are central to the research but not yet described in published literature, software must be made available to editors and reviewers. We strongly encourage code deposition in a community repository (e.g. GitHub). See the Nature Portfolio guidelines for submitting code & software for further information.

Data

Policy information about availability of data

All manuscripts must include a data availability statement. This statement should provide the following information, where applicable:

- Accession codes, unique identifiers, or web links for publicly available datasets
- A description of any restrictions on data availability
- For clinical datasets or third party data, please ensure that the statement adheres to our policy

Data of the participants of the Netherlands Twin Register cannot be made publicly available due to the EU's General Data Protection Regulation, but they are available for researchers via the Netherlands Twin Register data access procedure (https://ntr-data-request.psy.vu.nl/).

Research involving human participants, their data, or biological material

Policy information about studies with human participants or human data. See also policy information about sex, gender (identity/presentation), and sexual orientation and race, ethnicity and racism.

Reporting on sex and gender	Sex was recorded in the NTR based on participant self-report and coded as male or female. More specifically, participants are asked to check a box next to the statement 'Gender' (Dutch: 'geslacht'), with the labels "male" and "female". No data on gender identity were used in the present dataset. Sex was included as a covariate in all statistical models. The present study was not designed test sex-specific effects; therefore, analyses were not stratified by sex.
Reporting on race, ethnicity, or other socially relevant groupings	Not applicable.
Population characteristics	See below.
Recruitment	At the start of the NTR, a commercial 'birth felicitation' bureau visited parents of newborns at home and through city councils. Additional recruitment is done with the support of the Dutch Society of Parents of Multiples, through (online) newsletters, and events.<br>More information about NTR recruitment, see 10.1375/twin.5.5.401, 10.107/thg.2019.93, and 10.1017/thg.2012.118
Ethics oversight	All procedures performed were in accordance with the ethical standards of the institutional and/or national research committee and with the 1964 Helsinki declaration. Data collection was approved by the Central Ethics Committee on Research involving Human Subjects of the University Medical Centres Amsterdam. Signed informed consents were obtained from all participants included in NTR. No compensation was provided for participants.

Note that full information on the approval of the study protocol must also be provided in the manuscript.

Field-specific reporting

Please select the one below that is the best fit for your research. If you are not sure, read the appropriate sections before making your selection.

☐ Life sciences ☒ Behavioural & social sciences ☐ Ecological, evolutionary & environmental sciences

For a reference copy of the document with all sections, see nature.com/documents/nr-reporting-summary-flat.pdf

Behavioural & social sciences study design

All studies must disclose on these points even when the disclosure is negative.

Study description	The study involves longitudinal collected quantitative data of voluntary participants of the Netherlands Twin Register between 1986 and 2024.
Research sample	Multiple waves of the longitudinal database of the Netherlands Twin Register (NTR) were used with data collected through self-reported questionnaires at various time points since 1987. NTR is a population-based sample of twins and their families who register voluntarily to participate. Detailed information on NTR is described in earlier reports (Boomsma et al., 2002, 2006; Ligthart et al., 2019; van Beijsterveldt et al., 2013). The sample is not fully representative for the Dutch population.
Sampling strategy	The sample for the current study was selected using NTR participants with available wellbeing data at age 14-16, and available data on sociodemographic, health, and lifestyle factors in early adulthood. Characteristics of the study population are described in Table 1. About 58% of the study population are women. The mean (SD) age of the adolescents was 15.9 (1.8) years.<br><br>Among adults, questionnaires were paper and pencil until ~2009, when surveys where party distributed online as a pilot. Afterwards, all questionnaires were surveyed online. Among the participants younger than 18y, paper and pencil remained until ~2016. No researcher is present during any of the data collection. Since all data is collected prior to the research, researcher involved in the data collection were blind to study hypotheses.

Data collection	Adolescents in the NTR are invited to complete a self-report questionnaire, including three measures of wellbeing, at age 14, 16, and 18. From age 18 years onwards, every two to three years, NTR participants receive questionnaires to measure various sociodemographic, health, and lifestyle-related factors including BMI, physical activity, smoking habits, and wellbeing (Ligthart et al., 2019; van Beijsterveldt et al., 2013). Since questionnaires are filled out every two to three years, it was possible to select outcome variables based on the two age categories (20-25 and 25-35).
Timing	Adolescents were invited between 2004/05 and 2012/13. Questionnaires for adults have been gathered between 2004 and 2020: survey 8 between Sept. 2009 and Dec 2012, survey 10 between May 2013 and December 2016, and survey 14 between June 2019 and February 2020.
Data exclusions	Participants with only one out of three wellbeing measures available to calculate the teenage factor wellbeing score were excluded (n=1290).
Non-participation	In general, the response rate in NTR is estimated to be between 40 and 50%, see 10.1017/thg.2012.140 and 10.1017/thg.2012.118.
Randomization	The current study does not use randomization or participants in experimental groups. Relevant covariates have been included in the statistical models.

Reporting for specific materials, systems and methods

We require information from authors about some types of materials, experimental systems and methods used in many studies. Here, indicate whether each material, system or method listed is relevant to your study. If you are not sure if a list item applies to your research, read the appropriate section before selecting a response.

Materials & experimental systems

n/a	Involved in the study
☒	☐ Antibodies
☒	☐ Eukaryotic cell lines
☒	☐ Palaeontology and archaeology
☒	☐ Animals and other organisms
☒	☐ Clinical data
☒	☐ Dual use research of concern
☒	☐ Plants

Methods

n/a	Involved in the study
☒	☐ ChIP-seq
☒	☐ Flow cytometry
☒	☐ MRI-based neuroimaging

Plants

Seed stocks	*Report on the source of all seed stocks or other plant material used. If applicable, state the seed stock centre and catalogue number. If plant specimens were collected from the field, describe the collection location, date and sampling procedures.*
Novel plant genotypes	*Describe the methods by which all novel plant genotypes were produced. This includes those generated by transgenic approaches, gene editing, chemical/radiation-based mutagenesis and hybridization. For transgenic lines, describe the transformation method, the number of independent lines analyzed and the generation upon which experiments were performed. For gene-edited lines, describe the editor used, the endogenous sequence targeted for editing, the targeting guide RNA sequence (if applicable) and how the editor was applied.*
Authentication	*Describe any authentication procedures for each seed stock used or novel genotype generated. Describe any experiments used to assess the effect of a mutation and, where applicable, how potential secondary effects (e.g. second site T-DNA insertions, mosiacism, off-target gene editing) were examined.*
